# Tuning Intrinsic Spin Hall Effect in Platinum/Ferrimagnetic Insulator Heterostructure in Moderately Dirty Regime

**DOI:** 10.3390/nano13192721

**Published:** 2023-10-07

**Authors:** Tianhui Li, Lin Liu, Zehan Chen, Wei Jia, Jianxin Ye, Xudong Cai, Doudou Huang, Wanshan Li, Fukang Chen, Xinjun Li, Jiahao Chen, Boxi Dong, Hang Xie, Anyuan Pan, Chao Zhi, Hongyu An

**Affiliations:** 1 College of New Materials and New Energies, Shenzhen Technology University, Shenzhen 518118, China; 2110413006@stumail.sztu.edu.cn (T.L.); 2210412039@stumail.sztu.edu.cn (L.L.); 2210412040@stumail.sztu.edu.cn (Z.C.); jia.wei11@byd.com (W.J.); 202100304054@stumail.sztu.edu.cn (J.Y.); 202100304040@stumail.sztu.edu.cn (X.C.); 202100302084@stumail.sztu.edu.cn (W.L.); 202100302072@stumail.sztu.edu.cn (F.C.); 202100304071@stumail.sztu.edu.cn (X.L.); 202003020125@stumail.sztu.edu.cn (B.D.); 202003020114@stumail.sztu.edu.cn (H.X.); 2 College of Integrated Circuits and Optoelectronic Chips, Shenzhen Technology University, Shenzhen 518118, China; 202100303006@stumail.sztu.edu.cn (D.H.); 202100303076@stumail.sztu.edu.cn (J.C.); 3 Dealour Electric Co., Ltd., Jiaxing 314001, China; pananyuan@dealour.cn

**Keywords:** spintronics, spin Hall effect, ferrimagnetic insulator, spin–orbit torque

## Abstract

Studying the mechanisms of the spin Hall effect (SHE) is essential for the fundamental understanding of spintronic physics. By now, despite the intensive studies of SHE on heavy metal (HM)/metallic magnet heterostructures, the SHE on HM/ferrimagnetic insulator (FMI) heterostructures still remains elusive. Here, we study the mechanism of SHE in the Pt/Tm${}_{3}$Fe${}_{5}$O${}_{12}$ (TmIG) heterostructure. We first tune the crystallinity and resistivity of Pt by an annealing method, and then study the spin–orbit torque (SOT) in the tuned-Pt/TmIG devices. The SOT generation efficiency per unit electric field and spin Hall angle were obtained, which are insensitive to the annealing temperature. We further demonstrate that the intrinsic contribution in the moderately dirty regime is responsible for the SHE in our Pt/TmIG bilayer. Our study provides an important piece of information for the SHE in FMI-based spintronic physics.

## 1. Introduction

Since its first observation, the spin Hall effect (SHE) has been central in spintronics over the last decades [1,2,3]. Typically, in a heavy metal (HM)/magnet heterostructure, by applying a charge current in the HM layer, the SHE can generate a spin current in the perpendicular direction of the charge current [4,5,6,7,8,9,10,11]. The spin current is injected into the magnet, exerting spin torques that can control the magnetization of the magnet. Generally, the SHE originates from the extrinsic or the intrinsic mechanisms [3,12,13,14,15,16]. The contribution of the extrinsic scattering is induced by the spin-dependent scattering due to the impurities or defects in materials. On the other hand, the intrinsic contribution is induced by the Berry curvature and the band structure in the materials. Three different regimes were revealed in previous studies, which are superclean regime, moderately dirty regime and dirty regime [3,16]. Generally, the three regimes can be distinguished by the relationship between the spin Hall conductivity $\sigma_{\mathrm{SHE}}$ and electric conductivity $\sigma_{\mathrm{xx}}$: $\sigma_{\mathrm{SHE}}$∝$\sigma_{\mathrm{xx}}^{\gamma }$. In the superclean regime, the extrinsic contribution is dominant due to that it is strongly diminished by the carrier scattering. The skew-scattering plays a key role and results in $\sigma_{\mathrm{SHE}}$∝$\sigma_{\mathrm{xx}}$. In the moderately dirty regime, the intrinsic contribution is dominant, and $\sigma_{\mathrm{SHE}}$ barely changes with $\sigma_{\mathrm{xx}}$ since the carrier scattering time is sufficiently long [17]. While in the dirty regime, $\sigma_{\mathrm{SHE}}$ drastically decreases with $\sigma_{\mathrm{xx}}$, and the scaling exponent γ is typically larger than the unity since the carrier scattering time is short, which limits the interband excitation that governs the intrinsic SHE [18].

Studying the mechanisms of the SHE is essential for the fundamental understanding of spintronic physics, and diverse studies of the SHE have been reported in the HM/metallic magnet heterostructures [12,13,14,15,16,19,20]. The SHE can be tuned by changing the composition of alloys [13,21,22,23]. For instance, by changing the composition of the Au-Cu alloy, Musha et al. reported the observation of the crossover between the extrinsic and intrinsic SHE [13]. In their study, the resistivity of the Au${}_{1-x}$Cu${}_{x}$ alloy film can be effectively tuned by changing the concentration *x*, since the resistivity of Au is much lower than that of Cu. From the relationship between the spin Hall conductivity and the electric conductivity, it was interpreted that the skew scattering plays a dominant role in the extrinsic regime, and the intrinsic Berry curvature was responsible for the intrinsic contribution. The SHE can also be tuned by doping impurities, such as the MgO incorporation in Pt [19], oxygen incorporation in Pt [16] or Pd [14]. By precisely controlling the concentration of the impurities in the HM layer, the electric resistivity of the HM layer can be effectively tuned, and then the relationship between the spin Hall conductivity and the electric conductivity is obtained. Combined with the SHE theory, the behind mechanism can be verified. In Zhu et al.’s study [19], the resistivity of the Pt${}_{1-x}$(MgO)${}_{x}$ film was tuned by the MgO concentration *x*. They found that the spin Hall conductivity of Pt can be effectively tuned by the finely dispersed MgO impurities in the dirty regime. It was verified that the spin Hall conductivity rapidly decreases with shortening the carrier lifetime, while maintaining the band structure. Manipulating the lattice constant of Pt can also tune the SHE [15]. In Soya et al.’s study [15], the lattice constant of Pt was expanded by the nitrogen incorporation, resulting in the increase in the resistivity. Through varying the nitrogen concentration, the resistivity of Pt can be efficiently manipulated, and they found that the variation of the spin Hall conductivity of Pt was dominated by the change of the scattering time, rather than the band structure. The SHE can also be tuned by changing the measuring temperature due to the variation of the electric resistivity with the temperature [12]. In Sagasta et al.’s study [12], the resistivity of Pt was tuned by the measuring temperature. By tuning the resistivity in a wide range through temperature, they obtained the crossover between the moderately dirty and the superclean regimes of the SHE. These studies have provided important information to unveil the physical origins of the SHE, promoting the fundamental understanding of spintronics.

By now, most previous studies of the SHE were based on HM/metallic magnet heterostructures, and the SHE on HM/ferrimagnetic insulator (FMI) heterostructures still remain elusive. The FMI possesses a low damping property, long spin diffusion length and absence of Ohmic loss, which is very promising for the future memory and logic devices with high speed and low-energy consumption [24,25,26,27,28,29,30,31]. Moreover, although the charge current cannot flow in the FMI, its magnetization can be detected and manipulated in the HM/FMI heterostructures. The accumulated spin current at the HM/FMI interface can propagate into the FMI through magnons, and act as spin–orbit torques (SOTs) to control the FMI magnetization [26,27]. Therefore, to study the mechanism of the SHE in the HM/FMI heterostructures is important for the FMI-based spintronic physics.

In this work, we study the SHE in the Pt/Tm${}_{3}$Fe${}_{5}$O${}_{12}$ (TmIG) heterostructures. As a typical FMI, TmIG is a stable ceramic material with high melting temperature. Therefore, the variation of the HM layer has a negligible effect on the TmIG in the HM/TmIG bilayer. This advantage enables us to precisely tune the SHE in the Pt without concerning the influence induced by the metallic magnet in the conventional Pt/metallic magnet heterostructures. We first tune the crystallinity and resistivity of Pt through annealing method in vacuum, and then study the spin–orbit torque (SOT) generation in the Pt/TmIG devices. The SOT generation efficiency and spin Hall angle as a function of the annealing temperature are obtained. We further demonstrate that the intrinsic contribution in the moderately dirty regime is responsible for the SHE in our Pt/TmIG bilayer.

## 2. Materials and Methods

A TmIG film with thickness of 4 nm was deposited on a (111)-oriented Gd${}_{3}$Sc${}_{2}$Ga${}_{3}$O${}_{12}$ (GSGG) single crystal substrate at 750 ${}^{\circ }$C by magnetron sputtering from a TmIG target. Pure argon gas was applied for the deposition. The lattice constant of the GSGG substrate is 12.554 Å, which is larger than that of the TmIG (12.330 Å). Thus, the epitaxial growth of TmIG on GSGG can generate tensile strain in the TmIG lattice, which favors the perpendicular magnetic anisotropy. Then, the TmIG film was cooled down to the room temperature, and a Pt film with thickness of 4 nm was deposited on the TmIG surface without breaking the vacuum. The film thickness was controlled by the deposition time with a precalibrated deposition rate. A set of samples were fabricated simultaneously, and then annealed separately at different temperatures for 1 h in vacuum. Microstructure and the surface morphology of the films were characterized by the x-ray diffraction (XRD) with Cu K${}_{\alpha }$ irradiation and the atomic force microscopy (AFM), respectively. For the measurement of the anomalous Hall effect (AHE), we patterned the films into Hall bar shapes. All the measurements were conducted at room temperature.

## 3. Results and Discussion

**Figure 1:**
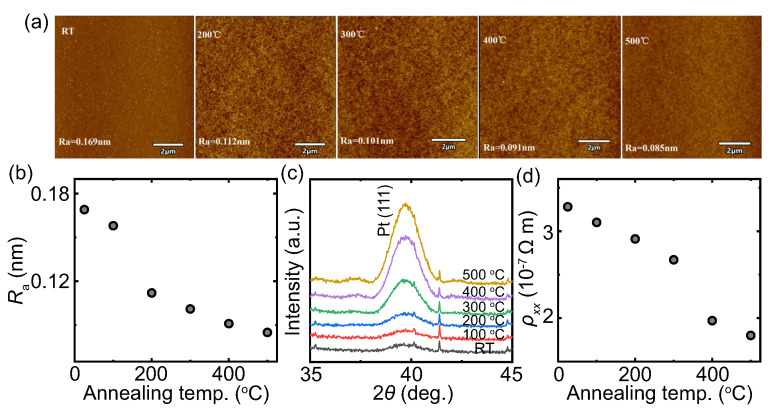
(**a**) AFM images of the surface morphology, (**b**) surface roughness, (**c**) XRD profiles and (**d**) resistivity for the Pt (4 nm)/TmIG (4 nm) bilayers annealed at different temperatures. The Hall bar devices were used for the resistivity measurement by conventional four-probe method.

Figure 1a shows the surface morphology of the Pt (4 nm)/TmIG (4 nm) films annealed at different temperatures. All the films exhibit continuous surfaces. The surface root-mean-square roughness (Figure 1b) in all the films is much lower than 1 nm, which decreases with the annealing temperature. The XRD profiles are shown in Figure 1c. All the films exhibit Pt (111) peak and the intensity increases with the annealing temperature. The AFM and XRD results indicate that the crystallinity of Pt becomes better by increasing the annealing temperature up to 500 ${}^{\circ }$C. This is further confirmed by the resistivity measurement. As shown in Figure 1d, the resistivity decreases from 3.3 × 10${}^{-7}$ Ω m to 1.8 × 10${}^{-7}$ Ω m by increasing the annealing temperature from room temperature (RT) to 500 ${}^{\circ }$C. The above results show that the annealing method can effectively tune the crystallinity and resistivity of Pt, which allows us to study the SHE of Pt in the Pt/TmIG bilayer by varying the Pt resistivity through annealing. It is considered that the annealing temperature up to 500 ${}^{\circ }$C has a minor effect on the TmIG layer, since the TmIG was deposited at 750 ${}^{\circ }$C. The much lower post-annealing temperature has a minor effect on improving the crystallinity of TmIG. We have confirmed that the saturation magnetization of TmIG is about 130 emu/cc, which has no change after annealing at 500 ${}^{\circ }$C. This is because even without annealing, perfect epitaxial growth with an atomically sharp interface between the TmIG and GSGG substrate has already been confirmed by the scanning transmission electron microscopy in our previous report [32].

**Figure 2:**
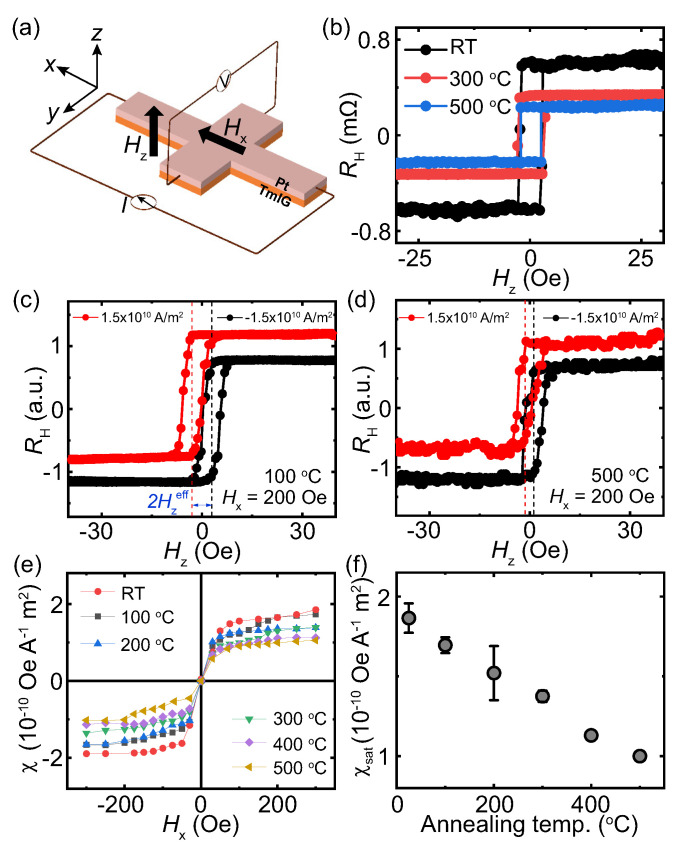
(**a**) Schematic of the setup for the AHE resistance measurement. (**b**) The typical AHE resistance curves of the Pt (4 nm)/TmIG (4 nm) devices. The SOT measurements for the devices (**c**) annealed at 100 ${}^{\circ }$C and (**d**) 500 ${}^{\circ }$C by sweeping $H_{z}$ with $H_{x}$ = 200 Oe. Slight vertical offsets for both AHE loops are introduced for clarity. (**e**) $H_{x}$ dependence of the SOT efficiency χ for different devices. (**f**) The maximum SOT efficiency $\chi_{\mathrm{sat}}$ as a function of the annealing temperature.

Figure 2a shows the schematic of the setup for the measurement of the AHE using the Hall bar devices. Square AHE hysteresis loops (see Figure 2b) were obtained in all the devices annealed at different temperatures. This result confirms that the TmIG has good perpendicular magnetic anisotropy in all the devices. Moreover, all the devices exhibit almost the same coercivity. This result further confirms that the post-annealing process at much lower temperature has a minor effect on the magnetic properties of TmIG. The AHE resistance decreases with the annealing temperature due to the decrease in the electric resistivity in Pt. For the SOT measurement, we used the method of the current-induced hysteresis loop shift, which is a well-established method for measuring SOT [33,34]. A constant magnetic field along the current direction $H_{x}$ was applied and the AHE curves were measured by sweeping $H_{z}$. The purpose for the application of $H_{x}$ is to break the symmetry of the SOT effective field. Therefore, the SOT effective field will act as an additional field in the out-of-plane direction and induce a shift in the AHE resistance curves. Figure 2c,d exhibit the typical shifted AHE curves with $H_{x}$ = 200 Oe for the devices annealed at 100 ${}^{\circ }$C and 500 ${}^{\circ }$C, respectively. By applying a charge current with a positive sign, the AHE curves shifts to the left, which is vice versa for the charge current with a negative sign. From the current-induced shift, the dampinglike torque effective field $H_{z}^{\mathrm{eff}}$ can be directly obtained. The dampinglike torque generation efficiency χ is calculated from χ = $H_{z}^{\mathrm{eff}}$/$J_{e}$, where $J_{e}$ is the current density. Figure 2e shows χ by changing $H_{x}$ for all the devices. $\chi_{\mathrm{sat}}$ is the maximum value obtained when it saturates at a sufficient $H_{x}$. $\chi_{\mathrm{sat}}$ gradually decreases by increasing the annealing temperature (Figure 2f). $\chi_{\mathrm{sat}}$ in the device without annealing is obtained to be 1.9 × 10${}^{-10}$ Oe A${}^{-1}$ m${}^{2}$, which decreases to 1.0 × 10${}^{-10}$ Oe A${}^{-1}$ m${}^{2}$ after annealing at 500 ${}^{\circ }$C.

**Figure 3:**
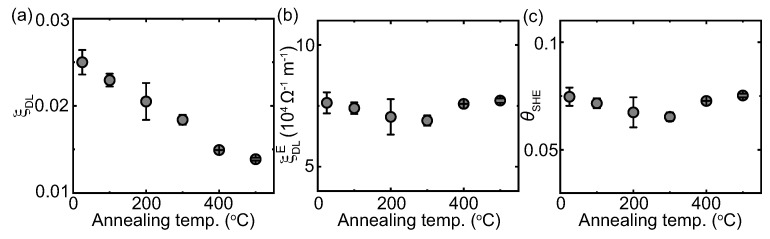
(**a**) The effective spin Hall angle $\xi_{\mathrm{DL}}$ by varying the annealing temperature, (**b**) the effective spin Hall angle per unit electric field $\xi_{\mathrm{DL}}^{E}$ = $\xi_{\mathrm{DL}}$/$\rho_{\mathrm{xx}}$ by varying the annealing temperature and (**c**) the spin Hall angle $\theta_{\mathrm{SHE}}$ = $\xi_{\mathrm{DL}}$/$T_{\mathrm{int}}$ by varying the annealing temperature. The error bars denote the propagated error from χ.

Based on the theory of the spin Hall effect, the effective spin Hall angle $\xi_{\mathrm{DL}}$ is calculated from the equation $\xi_{\mathrm{DL}}$ = $\chi_{\mathrm{sat}}$ $\left(\mu_{0}M_{s}t\right)$/$\left(\hbar /2e\right)$, where $M_{s}$, *t* and *ℏ* are the saturation magnetization, TmIG thickness and reduced Planck’s constant, respectively [34]. Figure 3a demonstrates the annealing temperature dependence of $\xi_{\mathrm{DL}}$, which gradually decreases with the annealing temperature. By increasing the annealing temperature from RT to 500 ${}^{\circ }$C, $\xi_{\mathrm{DL}}$ decreases from 0.025 to 0.014. The effective spin Hall angle per unit electric field $\xi_{\mathrm{DL}}^{E}$ = $\xi_{\mathrm{DL}}$/$\rho_{\mathrm{xx}}$ is plotted in Figure 3b, which is almost independent with the annealing temperature. The spin Hall angle is calculated from the equation $\theta_{\mathrm{SHE}}$ = $\xi_{\mathrm{DL}}$/$T_{\mathrm{int}}$. The interfacial spin transparency $T_{\mathrm{int}}$ is expressed as [19]

(1) $$T_{\mathrm{int}}=\left[1-\sec h(d/\lambda_{s})\right]/\left[1+G_{\mathrm{HM}}\tanh\left(d/\lambda_{s}\right)/2G_{\mathrm{HM}/\mathrm{FM}}^{↑↓}\right],$$

where *d*, $\lambda_{s}$, $G_{\mathrm{HM}}$ and $G_{\mathrm{HM}/\mathrm{FM}}^{↑↓}$ are the Pt thickness, spin diffusion length, spin conductance and spin mixing conductance at the Pt/TmIG interface, respectively. $\lambda_{s}$ is obtained using $\lambda_{s}$ = 0.77 × 10${}^{-15}$ Ω m${}^{2}$/$\rho_{\mathrm{xx}}$ [35], and $G_{\mathrm{HM}}$ is obtained by $G_{\mathrm{HM}}$ = $\sigma_{\mathrm{xx}}$/$\lambda_{s}$, where $\sigma_{\mathrm{xx}}$ is the electric conductivity of Pt [19]. For the device without annealing, $\lambda_{s}$ = 2.35 nm and $G_{\mathrm{Pt}}$ = 1.3 × 10${}^{15}$ $\Omega^{-1}$ m${}^{-2}$ are obtained. Combined with the experimental value $G_{\mathrm{Pt}/\mathrm{TmIG}}^{↑↓}$≈ 6.5 × 10${}^{14}$ $\Omega^{-1}$ m${}^{-2}$ [27], we obtain T${}_{\mathrm{int}}$ = 0.33 and $\theta_{\mathrm{SHE}}$ = 0.074. For the device annealed at 500 ${}^{\circ }$C, $\lambda_{s}$ is obtained to be 4.3 nm, T${}_{\mathrm{int}}$ is 0.18 and $\theta_{\mathrm{SHE}}$ is 0.075. Figure 3c shows $\theta_{\mathrm{SHE}}$ by changing the annealing temperature, which barely changes with the annealing temperature. The above results show that although the post-annealing process can improve the crystallinity and reduce the resistivity of Pt, the interfacial spin transparency at the Pt/TmIG interface decreases, which has a minor effect on the intrinsic spin Hall angle.

**Figure 4:**
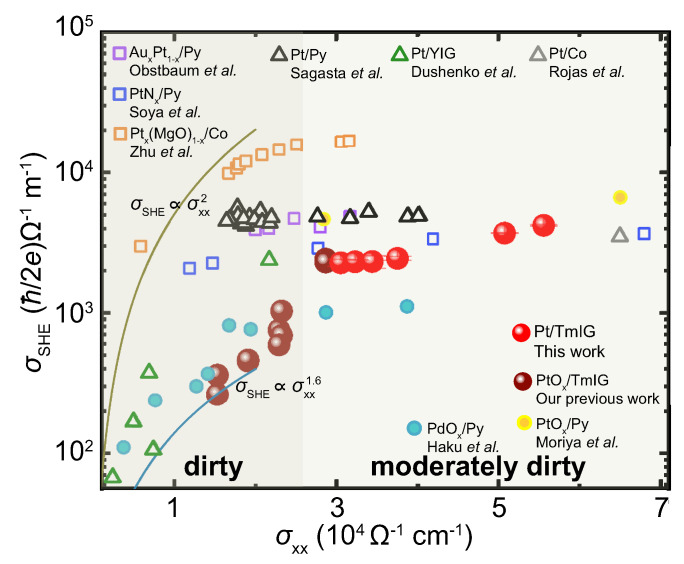
The spin Hall conductivity $\sigma_{\mathrm{SHE}}$ as a function of the electric conductivity $\sigma_{\mathrm{xx}}$. For comparison, the data in previous reports on Au${}_{x}$Pt${}_{1-x}$/Py [21], PtN${}_{x}$/Py [15], Pt${}_{x}$(MgO)${}_{1-x}$/Co [19], Pt/Py [12], Pt/YIG [36], Pt/Co [37], PdO${}_{x}$/Py [14], PtO${}_{x}$/Py [16] and PtO${}_{x}$/TmIG [38] were plotted. $\sigma_{\mathrm{SHE}}$∝$\sigma_{\mathrm{xx}}$${}^{1.6}$ and $\sigma_{\mathrm{SHE}}$∝$\sigma_{\mathrm{xx}}$${}^{2}$ are suggested by the AHE and SHE theories [3,39].

In the following, we analyze the SHE mechanism in our Pt/TmIG bilayers. The spin Hall conductivity $\sigma_{\mathrm{SHE}}$ is calculated using the equation $\sigma_{\mathrm{SHE}}$ = $\sigma_{\mathrm{xx}}$(ℏ/2e)$\theta_{\mathrm{SHE}}$. Figure 4 demonstrates the $\sigma_{\mathrm{SHE}}$ by varying the electric conductivity $\sigma_{\mathrm{xx}}$. The data from previous reports have also been added for comparison. As can be seen, although different materials and different measuring methods from previous reports were used, the relationship between $\sigma_{\mathrm{SHE}}$ and $\sigma_{\mathrm{xx}}$ can be clearly observed. Below about 2.6 × 10${}^{4}$ $\Omega^{-1}$ cm${}^{-1}$, $\sigma_{\mathrm{SHE}}$ drastically decreases by decreasing $\sigma_{\mathrm{xx}}$. Above 2.6 × 10${}^{4}$ $\Omega^{-1}$ cm${}^{-1}$, $\sigma_{\mathrm{SHE}}$ is insensitive with $\sigma_{\mathrm{xx}}$. In our study, we can exclude the extrinsic contribution because the extrinsic scattering only plays a significant role when the electric conductivity is larger than 6 × 10${}^{4}$ $\Omega^{-1}$ cm${}^{-1}$ [12]. For the intrinsic mechanism, the spin Hall conductivity varies differently in the moderately dirty and dirty regimes. In the moderately dirty regime, the carrier scattering time is sufficiently long, and the spin Hall conductivity is insensitive to the scattering. While in the dirty regime, the spin Hall conductivity decreases with decreasing the scattering time since the carrier scattering time is short. In Figure 4, compared with previous reports, The minor change of $\sigma_{\mathrm{SHE}}$ with $\sigma_{\mathrm{xx}}$ is consistent with the intrinsic SHE scenario, which confirms that the intrinsic contribution in the moderately dirty regime is responsible for the SHE in our Pt/TmIG bilayer.

## 4. Conclusions

To summarize, the SHE in the Pt/TmIG heterostructure has been studied by controlling the crystallinity and resistivity of Pt through the annealing method. The SOT generation efficiency and the spin Hall angle were obtained in the Pt/TmIG devices after annealing at different temperatures. Our study shows that the spin Hall angle is insensitive to the annealing temperature. We further demonstrate that the intrinsic contribution in the moderately dirty regime is responsible for the SHE in our Pt/TmIG bilayer. Our study provides an important piece of information for the SHE in FMI-based spintronic physics.

## Data Availability

All the data present in this paper will be made available upon reasonable request. Please contact the corresponding author for further information.
